# Supplementary material for: HiChIP-Peaks: a HiChIP peak calling algorithm
Source: Bioinformatics. 2020 Mar 24;36(12):3625–31. doi: 10.1093/bioinformatics/btaa202 (PMC7320601; doi:10.1093/bioinformatics/btaa202)

HiChIP-Peaks: A HiChIP peak calling algorithm

Chenfu Shi, Magnus Rattray and Gisela Orozco

Supplementary tables and figures

Table S1

Number of reads that contain uncut restriction sites within HiChIP datasets. Approximately 15 million out of the 98 million reads from the dataset contain an uncut MboI restriction site motif. In dark blue are the 6bp motifs that could have been generated by the re-ligation event. All other motifs are evidence of uncut restriction sites. Dataset from Naïve T cells, Biological replicate 2, technical replicate 1, read 1 (Fwd).

Accession number	Reads	
SRR5831497 (Fwd)	98201048	Approximately 15 million uncut restriction sites on R1

Motif	counts	Motif	counts
AGATCA	1139756	TGATCA	840523
AGATCC	738643	TGATCC	1062812
AGATCT	875481	TGATCT	1167673
AGATCG	12627791	TGATCG	11874109
CGATCA	11220492	GGATCA	1154531
CGATCC	10110047	GGATCC	660955
CGATCT	11952588	GGATCT	741551
CGATCG	3949528	GGATCG	11027058

Table S2

Motifs identified as significantly enriched from differentially bound peaks. Results from HOMER using differentially bound peaks from the specified contrasts.

Tregs vs Naïve T cells

Motif Name	P-value	Log P-value	q-value (Benjamini)
ISRE(IRF)/ThioMac-LPS-Expression(GSE23622)	1.00E-21	-5.00E+01	0
IRF2(IRF)/Erythroblas-IRF2-ChIP-Seq(GSE36985)	1.00E-17	-4.06E+01	0
IRF1(IRF)/PBMC-IRF1-ChIP-Seq(GSE43036)	1.00E-10	-2.33E+01	0
Ets1-distal(ETS)/CD4+-PolII-ChIP-Seq(Barski_et_al.)	1.00E-08	-1.85E+01	0
Bach1(bZIP)/K562-Bach1-ChIP-Seq(GSE31477)	1.00E-06	-1.45E+01	0
NF-E2(bZIP)/K562-NFE2-ChIP-Seq(GSE31477)	1.00E-06	-1.39E+01	0.0001
ETS:RUNX(ETS,Runt)/Jurkat-RUNX1-ChIP-Seq(GSE17954)	1.00E-05	-1.34E+01	0.0001
Nrf2(bZIP)/Lymphoblast-Nrf2-ChIP-Seq(GSE37589)	1.00E-04	-1.09E+01	0.0008
Jun-AP1(bZIP)/K562-cJun-ChIP-Seq(GSE31477)	1.00E-03	-8.39E+00	0.0088
E2F(E2F)/Hela-CellCycle-Expression	1.00E-03	-7.29E+00	0.024
RFX(HTH)/K562-RFX3-ChIP-Seq(SRA012198)	1.00E-03	-6.92E+00	0.0318
NFkB-p65-Rel(RHD)/ThioMac-LPS-Expression(GSE23622)	1.00E-02	-6.75E+00	0.035

Th17 vs Naïve T cells

Motif Name	P-value	Log P-value	q-value (Benjamini)
**ISRE(IRF)/ThioMac-LPS-Expression(GSE23622)**	1.00E-11	-2.66E+01	0
**IRF2(IRF)/Erythroblas-IRF2-ChIP-Seq(GSE36985)**	1.00E-09	-2.29E+01	0
**ETS:RUNX(ETS,Runt)/Jurkat-RUNX1-ChIP-Seq(GSE17954)**	1.00E-08	-1.85E+01	0
**Ets1-distal(ETS)/CD4+-PolII-ChIP-Seq(Barski_et_al.)**	1.00E-08	-1.84E+01	0
**NFkB-p65-Rel(RHD)/ThioMac-LPS-Expression(GSE23622)**	1.00E-04	-1.14E+01	0.0008
**RFX(HTH)/K562-RFX3-ChIP-Seq(SRA012198)**	1.00E-04	-1.01E+01	0.0022
**Bach1(bZIP)/K562-Bach1-ChIP-Seq(GSE31477)**	1.00E-04	-9.50E+00	0.0036
**IRF1(IRF)/PBMC-IRF1-ChIP-Seq(GSE43036)**	1.00E-03	-9.17E+00	0.0045
**Nrf2(bZIP)/Lymphoblast-Nrf2-ChIP-Seq(GSE37589)**	1.00E-03	-7.84E+00	0.0152
**Rfx2(HTH)/LoVo-RFX2-ChIP-Seq(GSE49402)**	1.00E-03	-7.45E+00	0.0205
**NF-E2(bZIP)/K562-NFE2-ChIP-Seq(GSE31477)**	1.00E-03	-7.12E+00	0.026
**T1ISRE(IRF)/ThioMac-Ifnb-Expression**	1.00E-02	-6.63E+00	0.0394

Figure S1

Reads are located around the restriction site, as expected from a Hi-C library. A) Distribution of reads' distance from closest restriction site. Dataset from Naïve T cells, Biological replicate 1, technical replicate 1. B-C) Pileup of short-range reads from HiChIP dataset Naïve T cells combined (using FitHiChIP's utility). The signal is more intense around the restriction sites, creating sparsity elsewhere which can bias other peak calling methods resulting in many small peaks. Hichipper attempts to compensate this by extending the peaks to the nearest fragment. HiChIP-peaks is based on the re-ligation site and doing so ignores the sparsity by design and maximises usable information. Data shown is from GM12878.

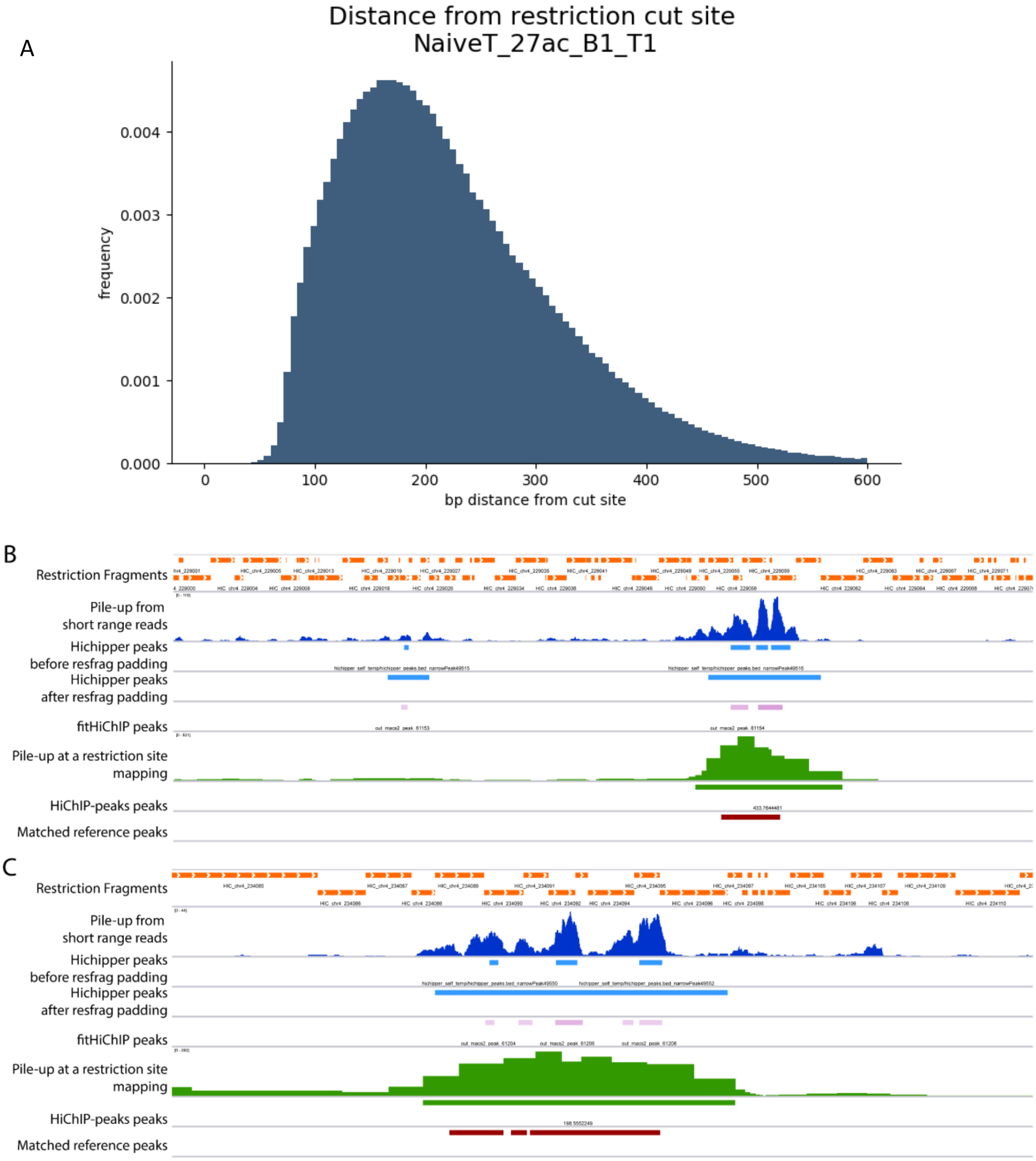

Figure S2

Proportion of read pairs identified as dangling ends, self-circle and re-ligation from the Naïve T cells dataset and a Hi-C library generated with the Arima-Hi-C kit (unpublished). Libraries generated with the Arima-Hi-C kit have almost no dangling ends and self-circle reads that can be used by Hichipper in SELF mode.

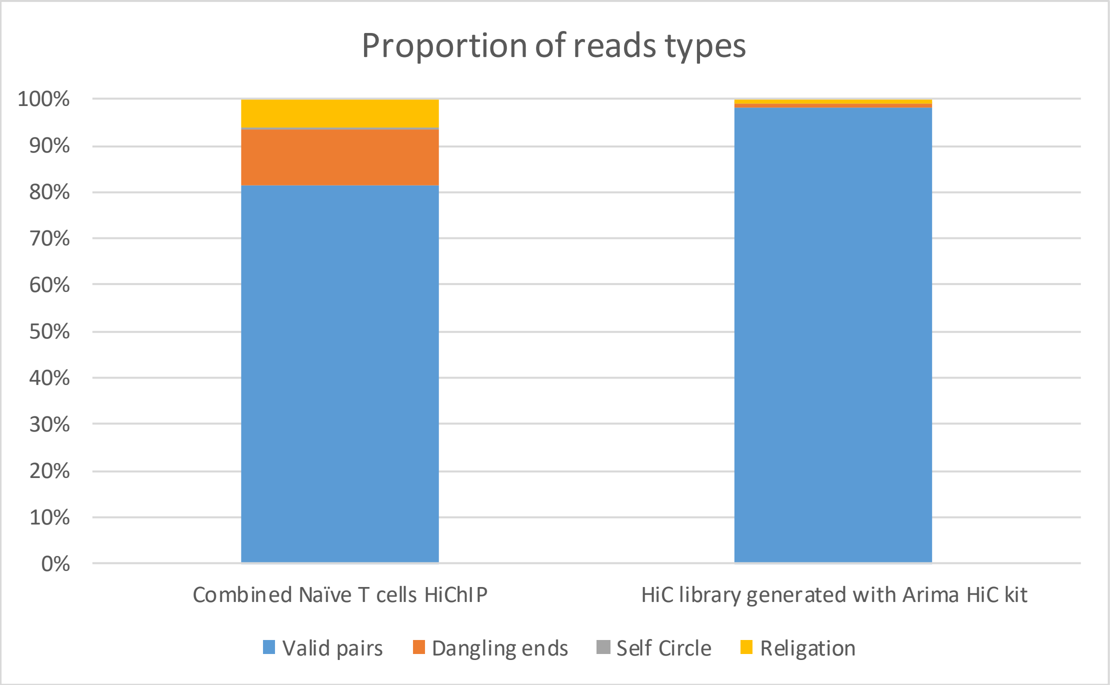

Figure S3

Read count distribution per restriction site as used for HiChIP-Peaks. The noise signal from the HiChIP datasets resembles a negative binomial distribution. Data from the combined Naïve T cells dataset.

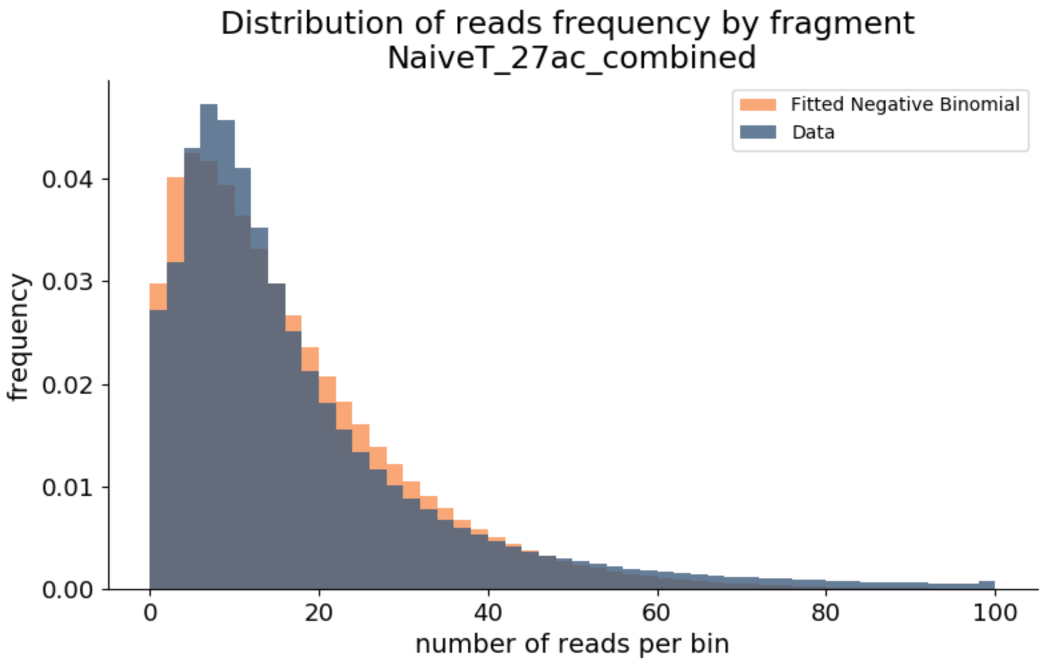

Figure S4

Fragment size bias fitted using a LOWESS fit. Fragment size is the sum of the fragments within the tested re-ligation sites. We use this fit to correct the expected background by fragment size in the negative binomial test. Data from the combined Naïve T cells dataset.

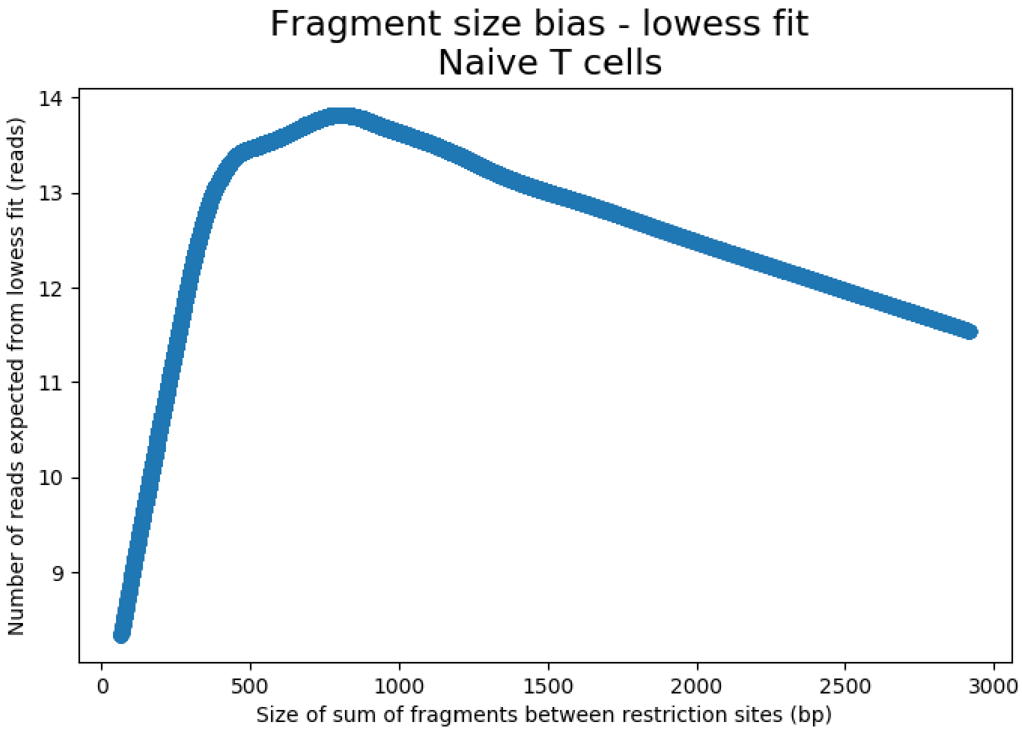

Figure S5

P-value distribution from negative binomial test. The null distribution is uniform, showing an appropriate fit of the background model. Data from the combined Naïve T cells dataset.

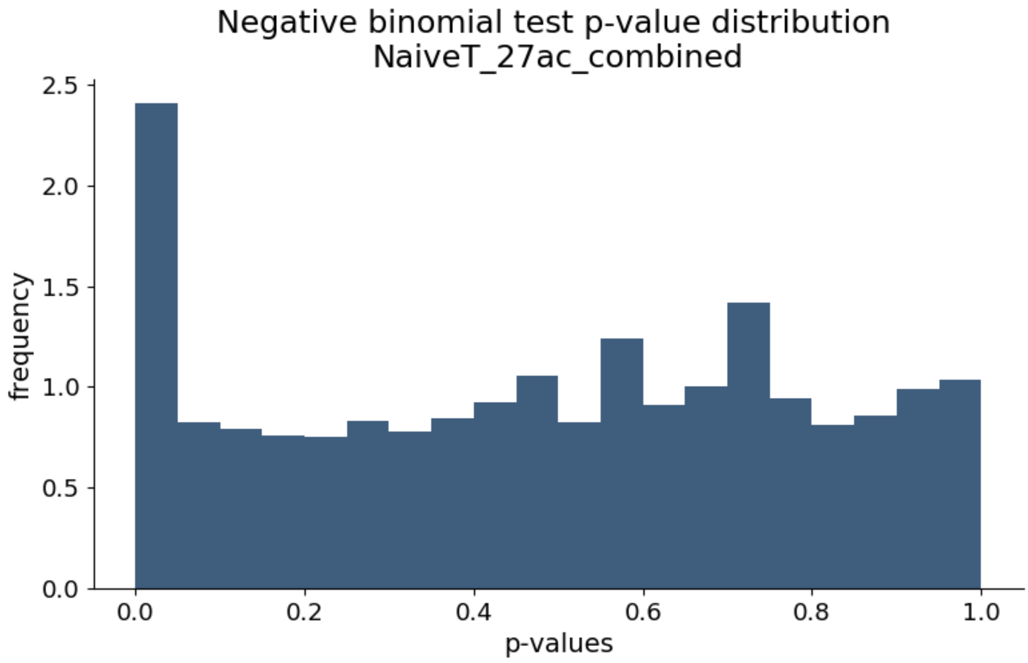

Figure S6

Peaks recalled from reference vs peaks called from Naïve T cells (A) and GM12878 (B). Our algorithm can identify more peaks from the reference while calling fewer peaks.

A

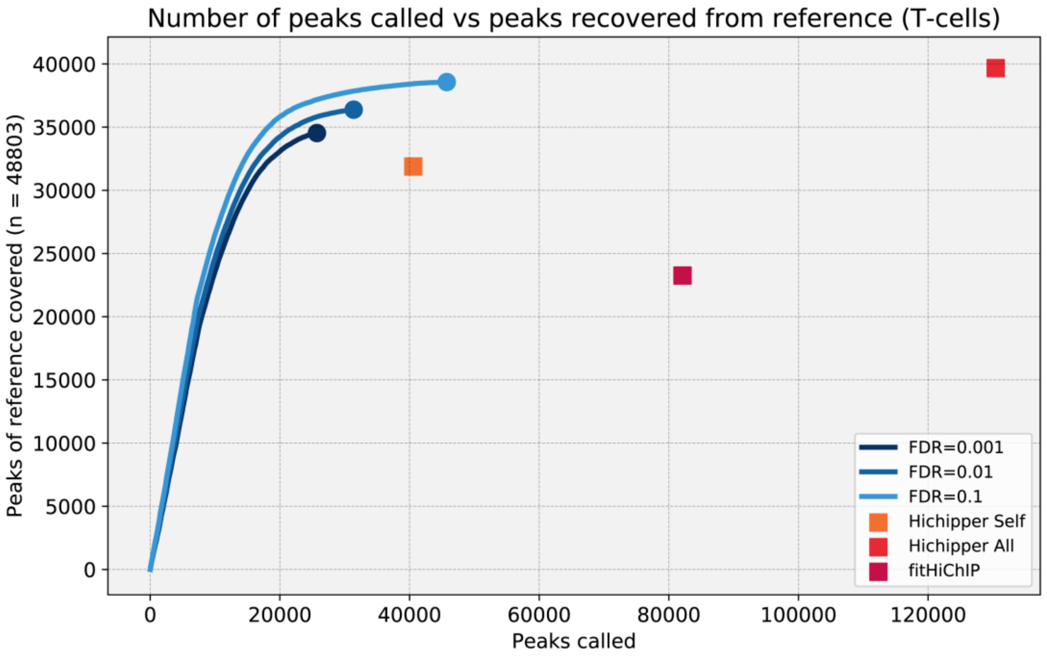

B

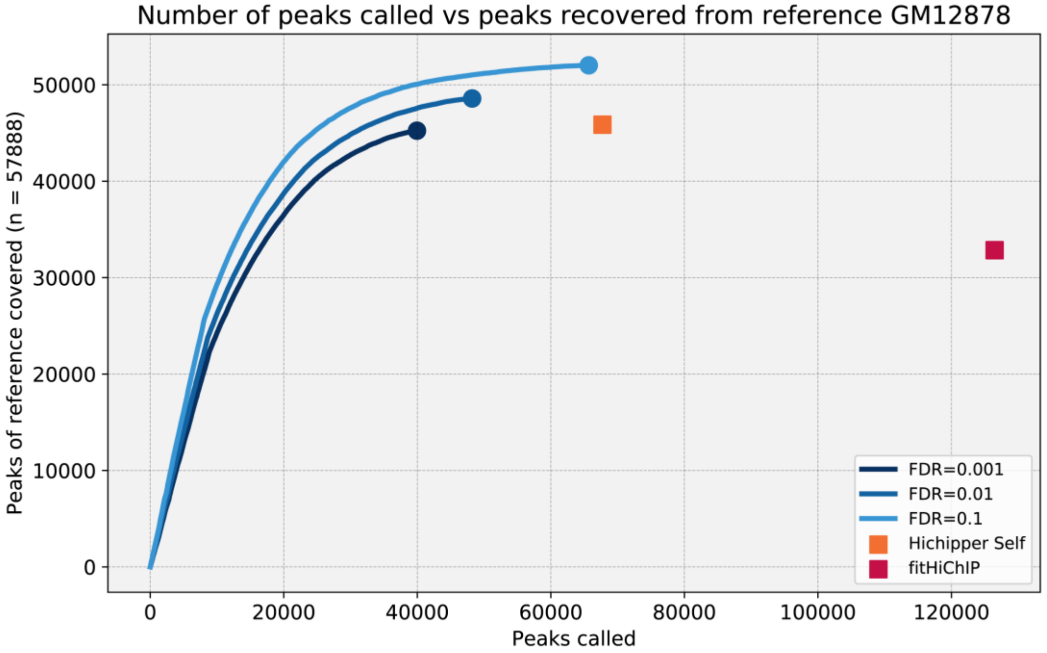

Figure S7

Peaks recalled from reference vs genome covered from Naïve T cells (A) and GM12878 (B). Even though the peaks identified by our algorithm can be larger than Hichipper's we can still identify more peaks from the reference at the same amount of genome covered when FDR is set at less than 0.01. FitHiChIP fares unfairly well in this metric because it produces a lot of small peaks that are dispersed along the genome.

A

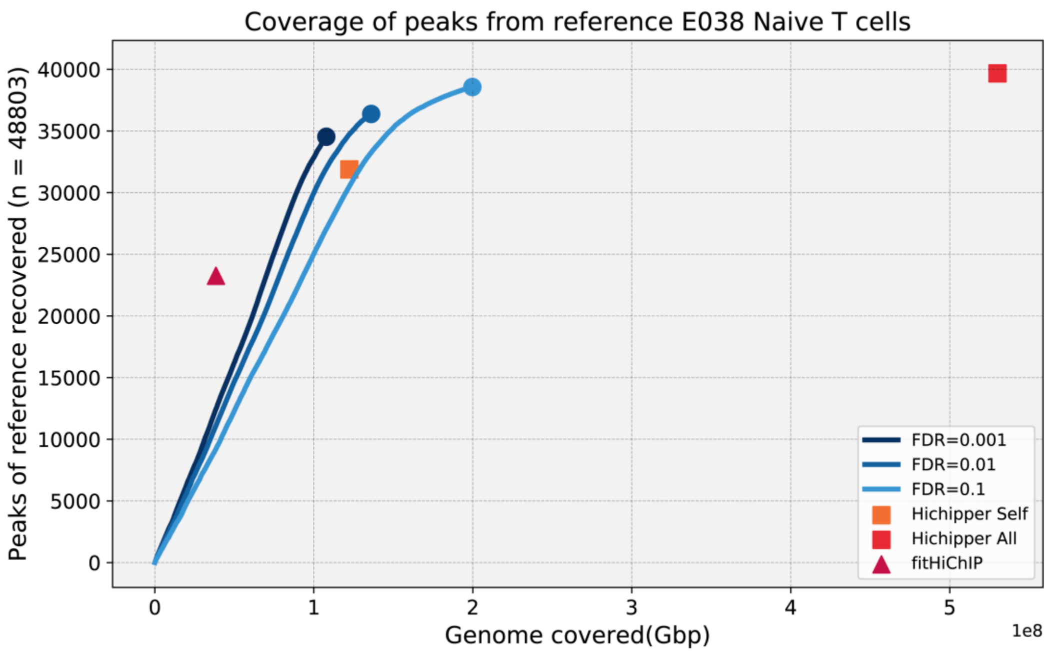

B

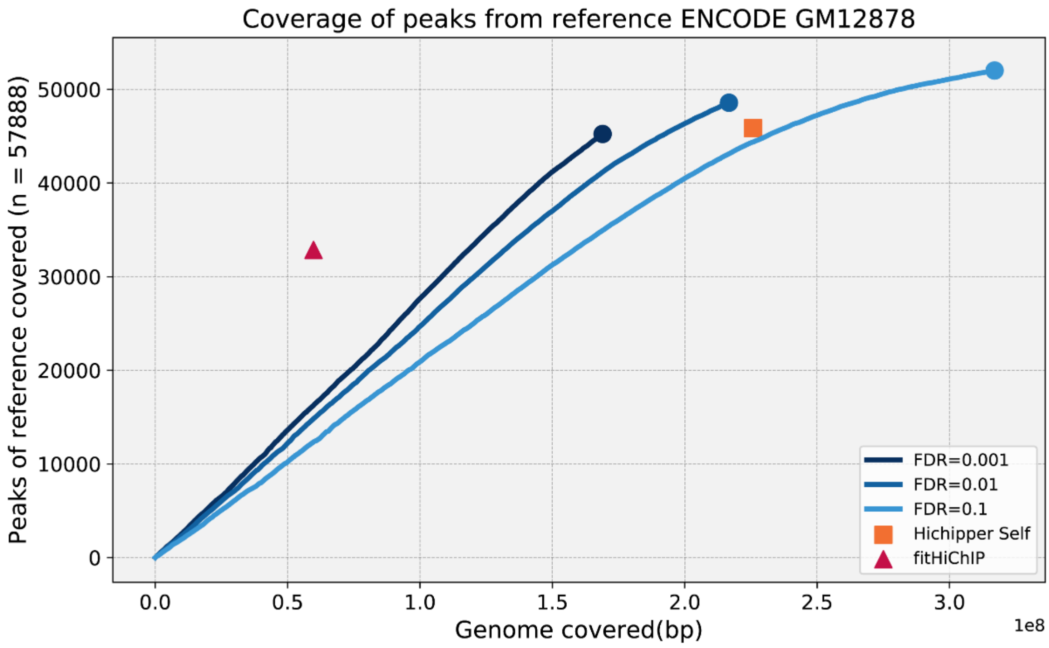

Figure S8

Precision-recall values for peak calling in subsampling analysis (vs full dataset). Our peak calling method is significantly more consistent compared to hichipper when read depth is reduced. (A) CD4+ Naïve T cells. (B) GM12878 cells.

A

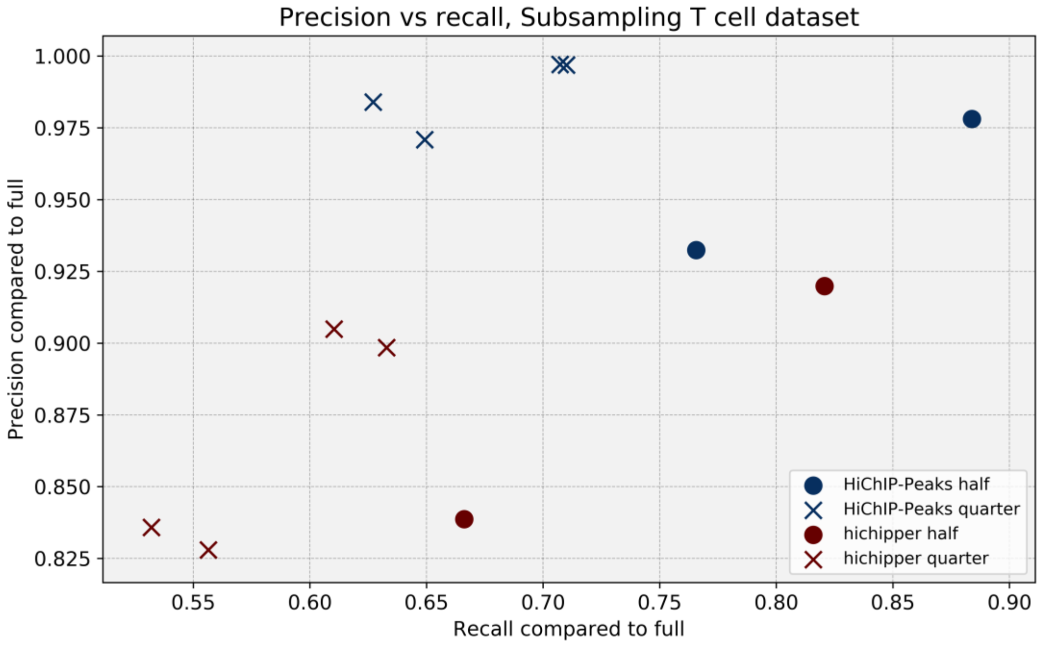

B

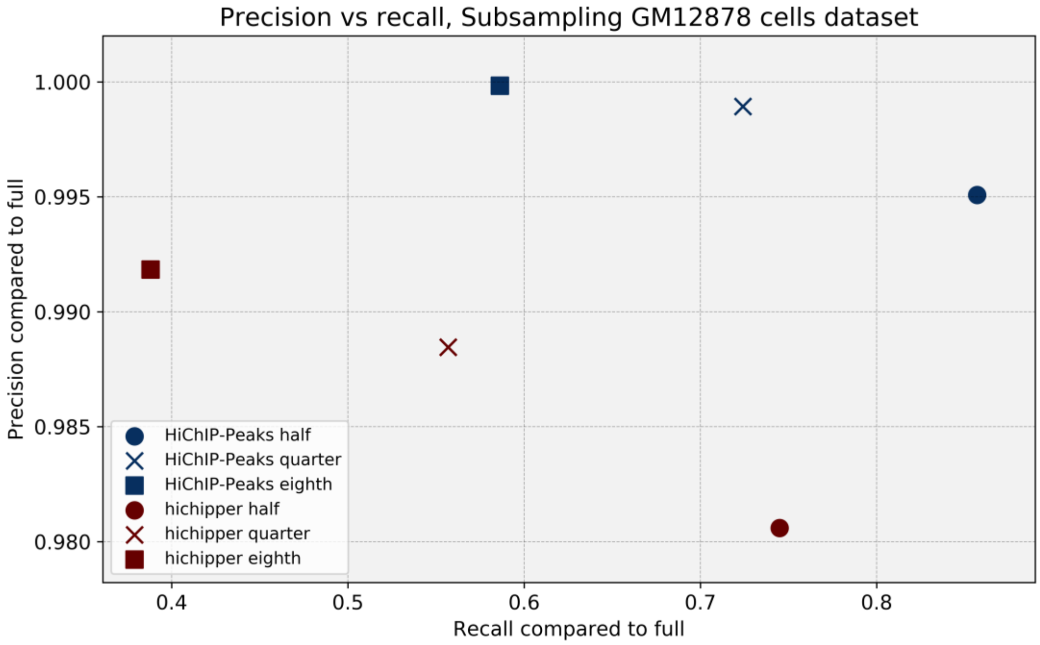

Figure S9

Overlap analysis between loops called using full depth datasets and subsampled datasets for CD4+ Naïve T cells. Supplying our peaks to Hichipper increases the recall of the loops identified from the full dataset without significant degradation in precision. (A) recall vs number of loops called. (B) precision-recall plot.

A

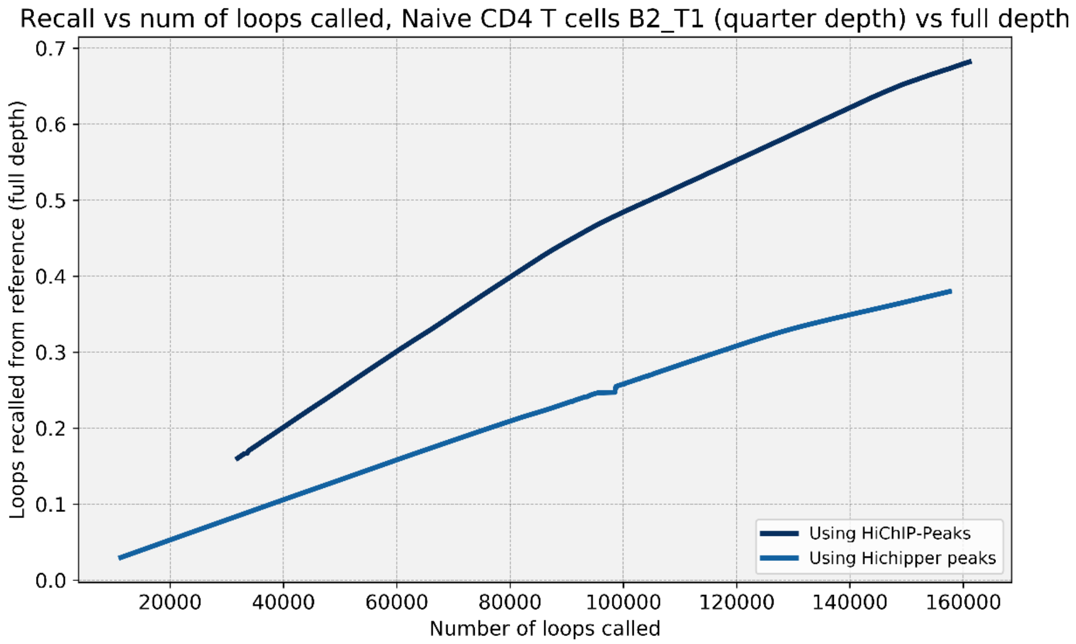

B

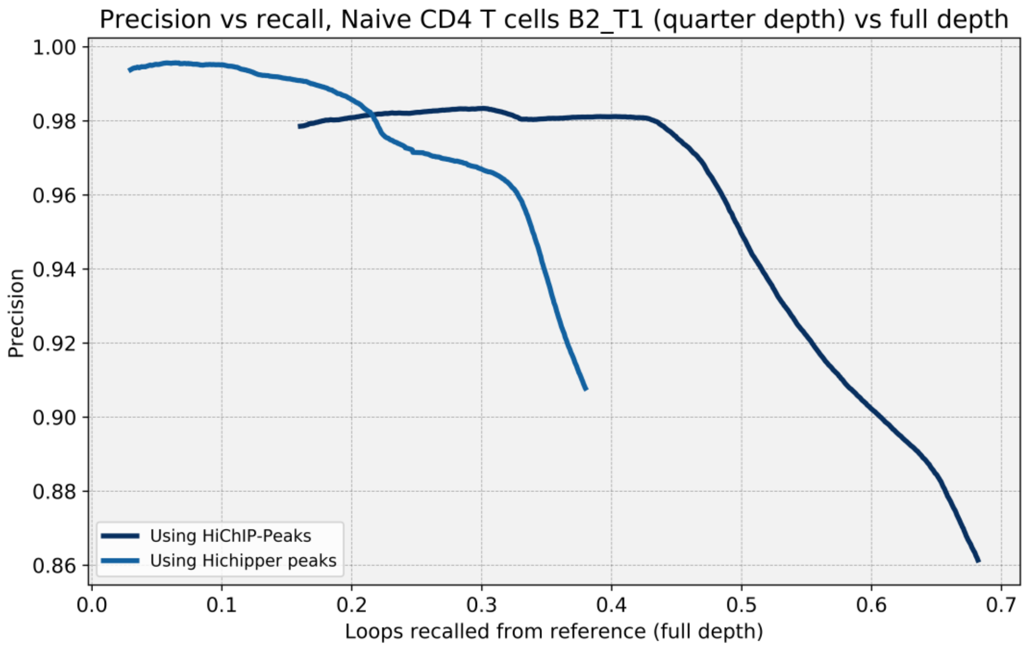

Figure S10

Overlap analysis between loops called using full depth datasets and subsampled datasets for GM12878 cells. Supplying our peaks to Hichipper increases the recall of the loops identified from the full dataset without significant degradation in precision. (A) recall vs number of loops called. (B) precision-recall plot.

A

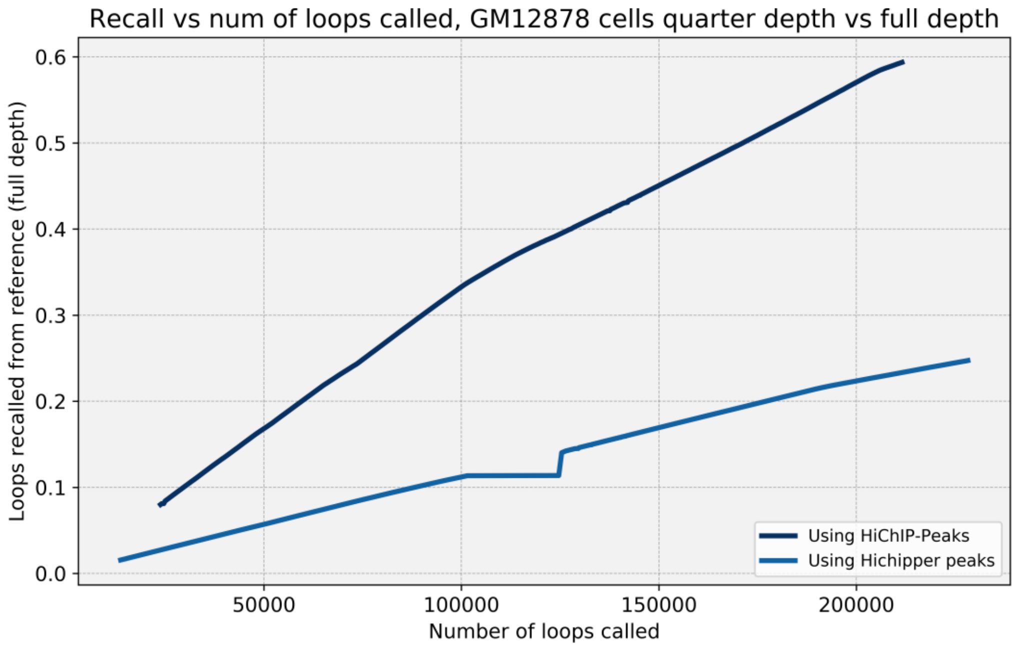

B

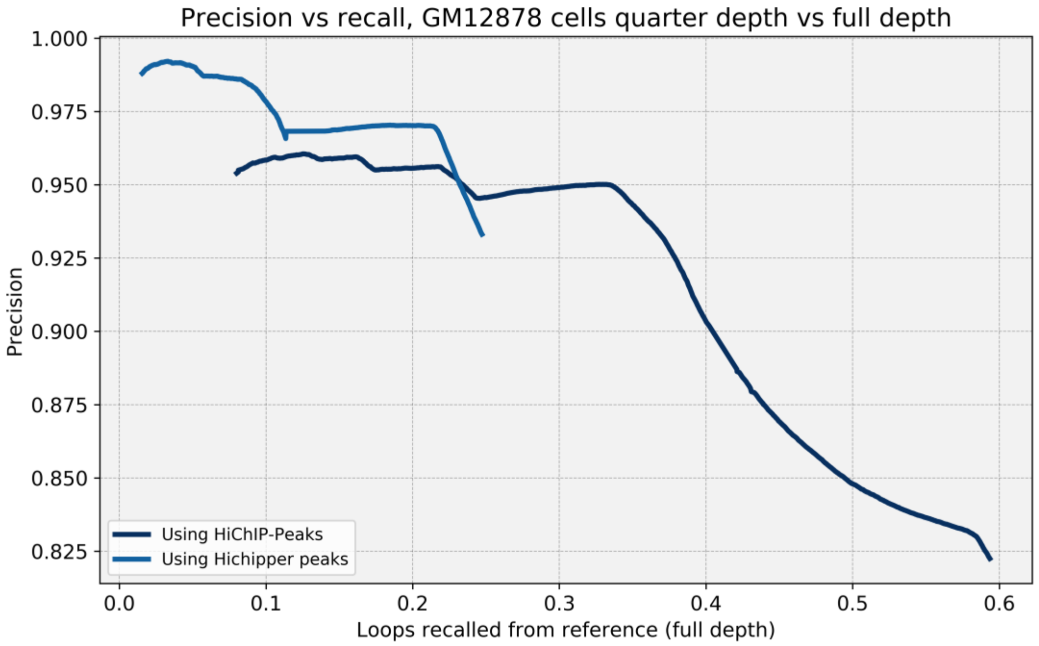

Figure S11

Overlap analysis between reference promoter capture Hi-C and Hichipper with default peaks and with our peaks in CD4+ Naïve T cells. Using our peaks to run Hichipper provides a higher recall at the same number of loops called (A) and with a higher precision (B) than using the default peaks.

A

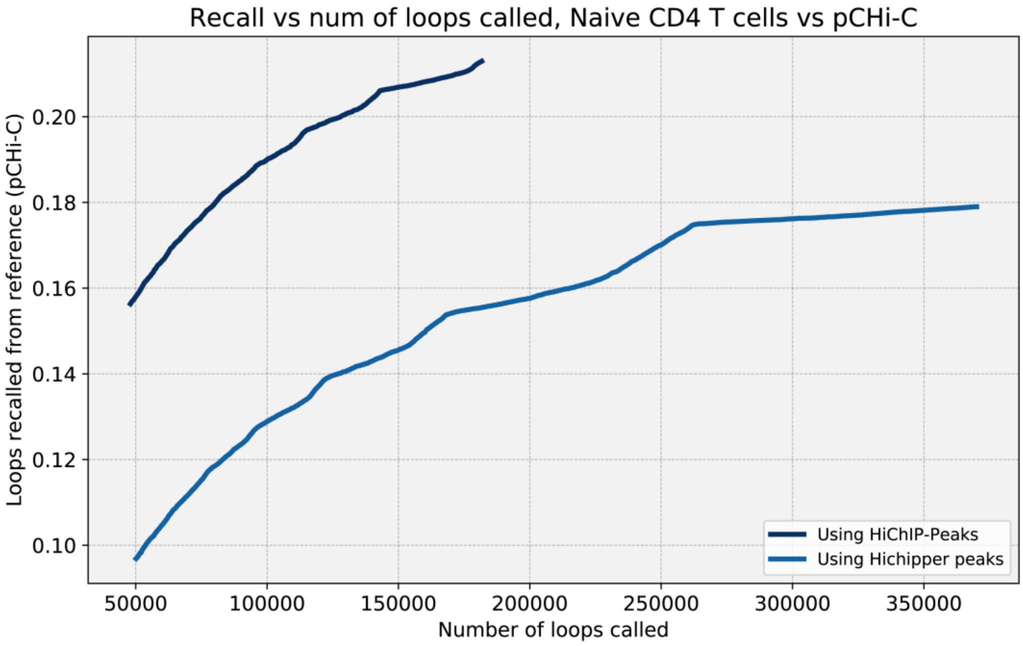

B

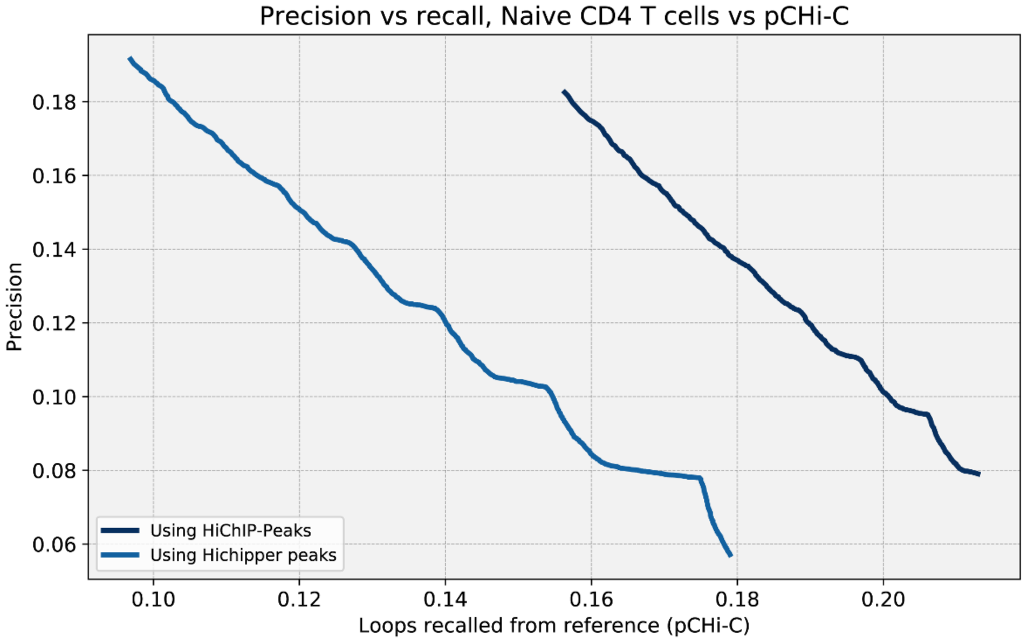

Figure S12

Overlap analysis between reference promoter capture Hi-C and Hichipper with default peaks and with our peaks in GM12878 cells. Using our peaks to run Hichipper provides a higher recall at the same number of loops called (A) and with a higher precision (B) than using the default peaks.

A

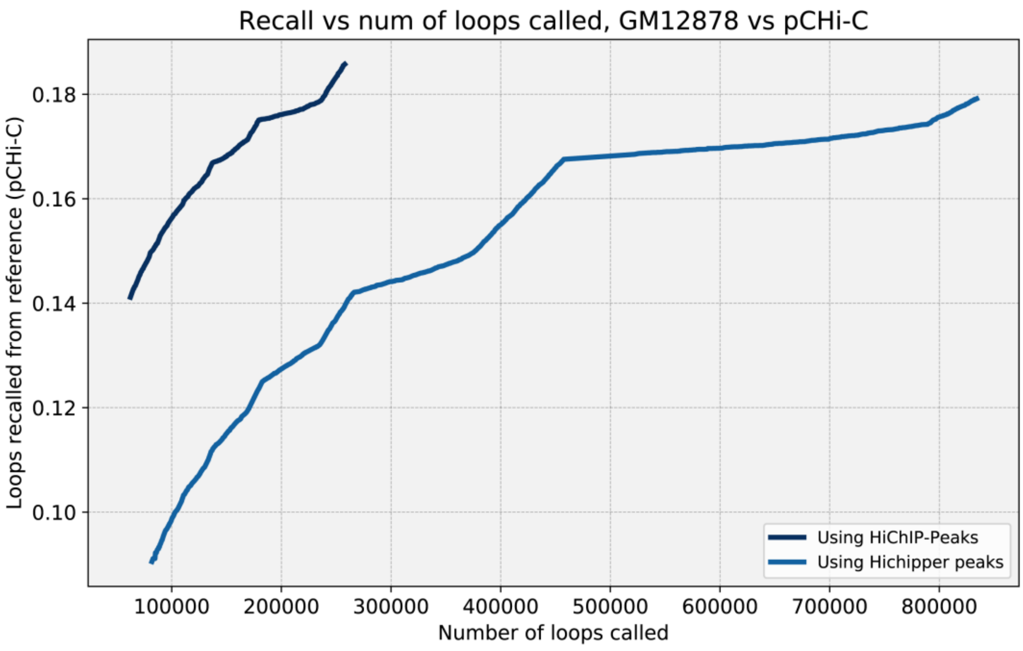

B

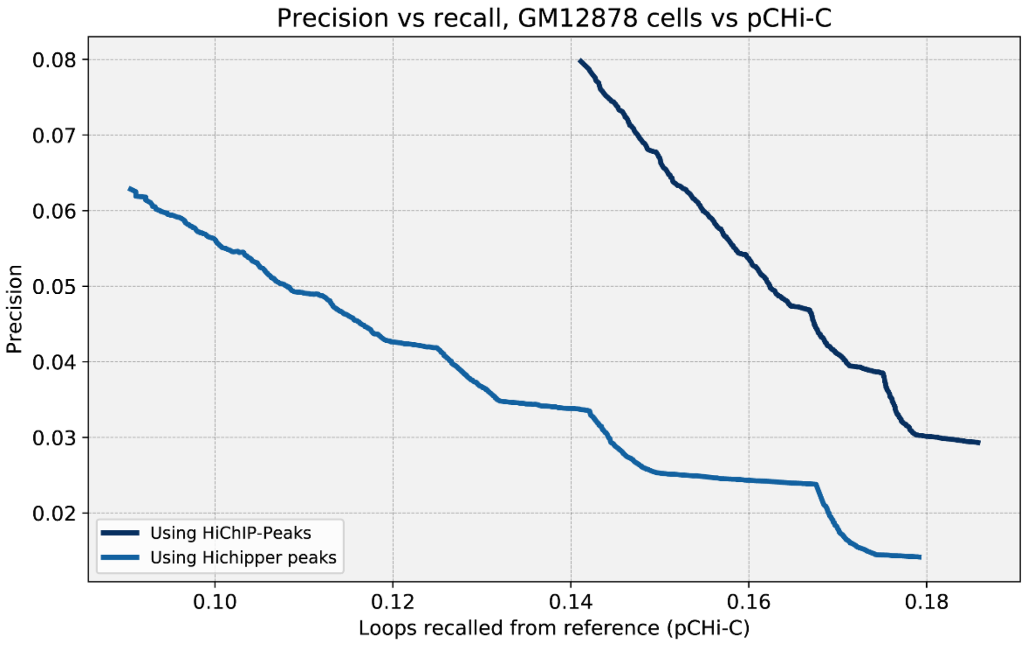

Figure S13

Overlap analysis between reference H3K27ac ChIA-PET and Hichipper with default peaks and with our peaks in K562 cells. Using our peaks to run Hichipper provides a higher recall at the same number of loops called (A) and with a higher precision (B) than using the default peaks.

A

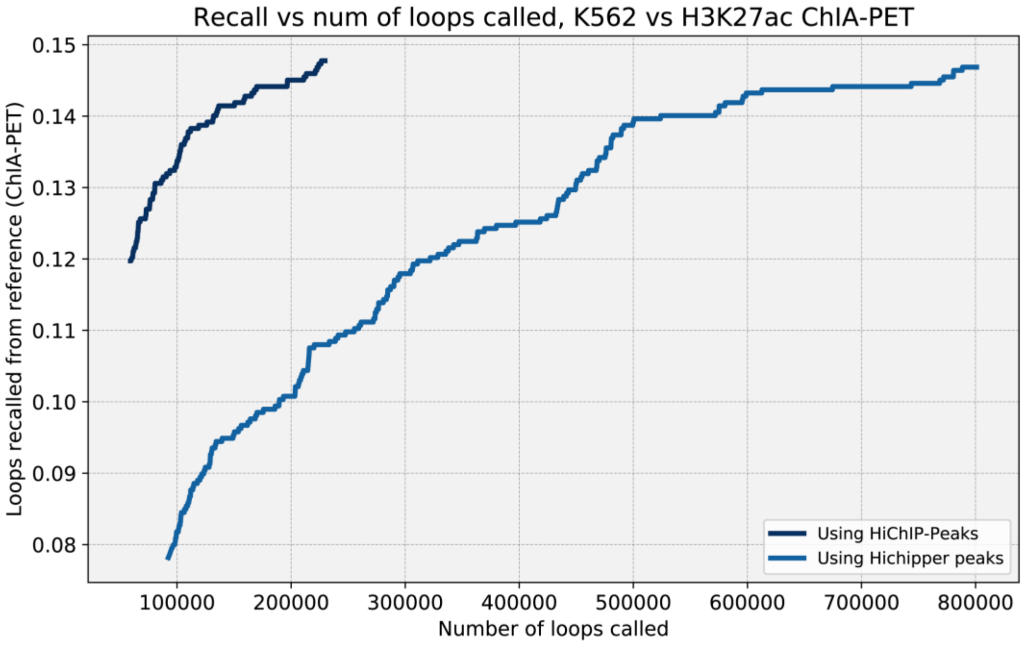

B

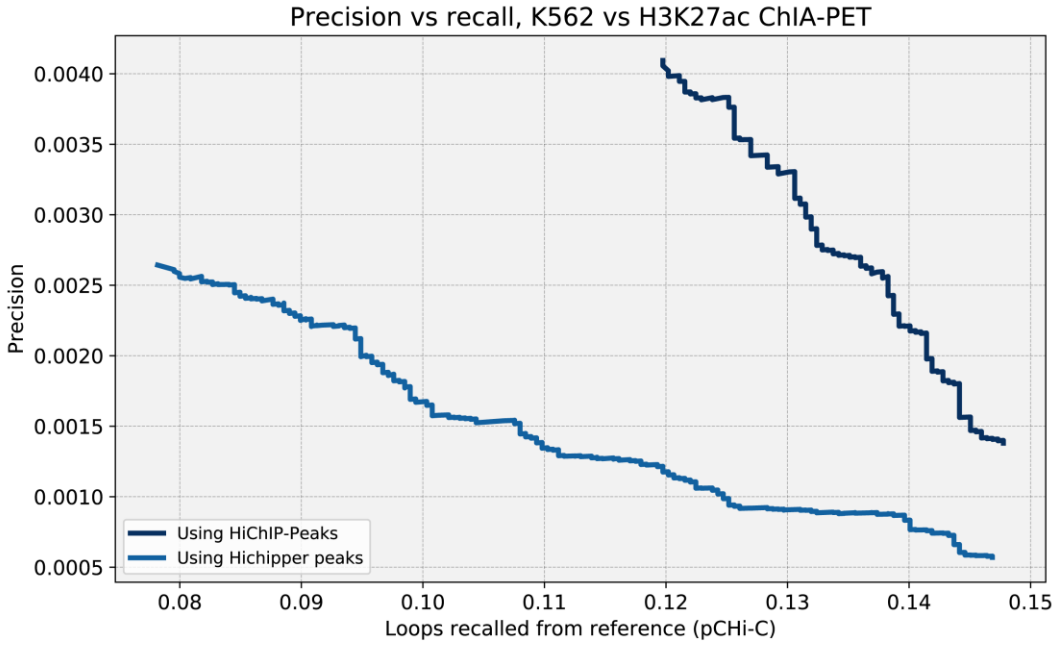

Figure S14

Comparing genome covered by loops with recall between reference promoter capture Hi-C and Hichipper with default peaks and with our peaks in CD4+ T cells (A) and GM12878 cells (B). The anchors generate using peaks from our software are larger due to the larger size of the anchors. We show that even comparing the total basepairs covered by anchors with recall we still have a better ratio.

A

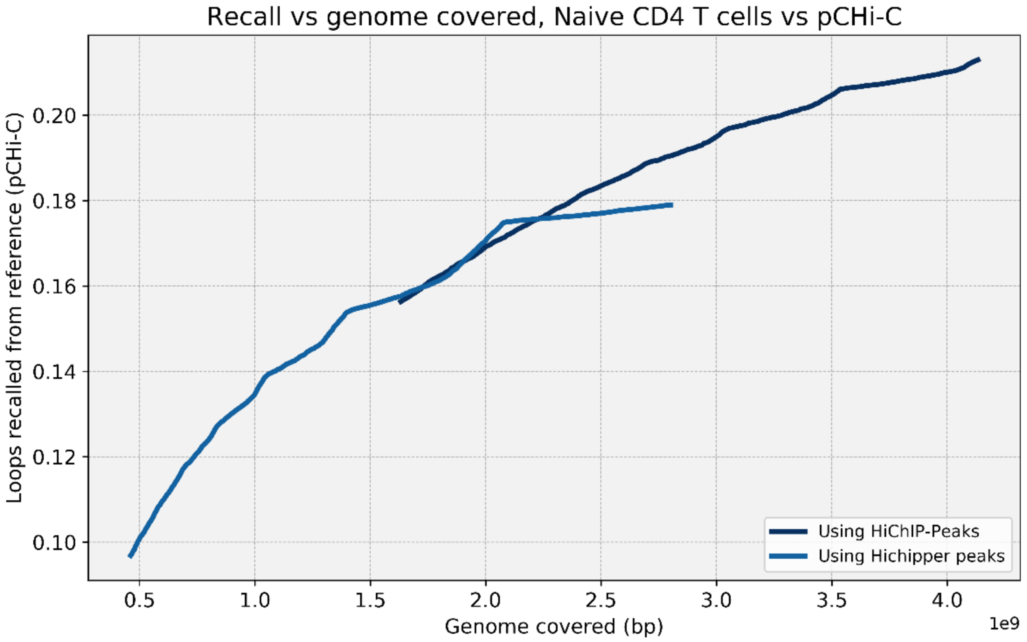

B

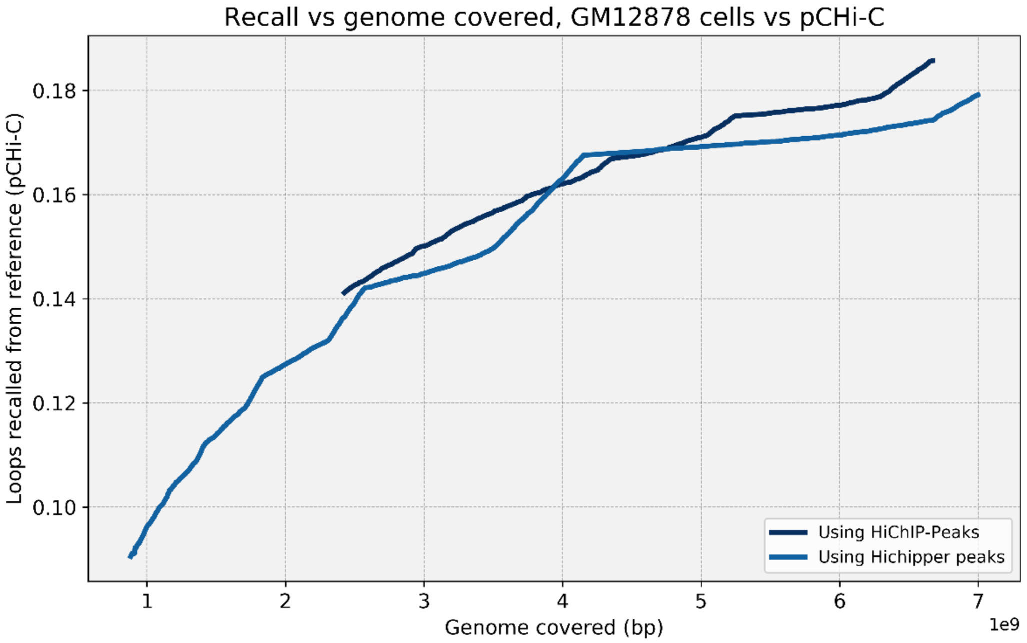

Figure S15

Comparing genome covered by loops with recall between H3K27ac ChIA-PET and Hichipper with default peaks and with our peaks in K562 cells. The anchors generate using peaks from our software are larger due to the larger size of the anchors. We show that even comparing the total basepairs covered by anchors with recall we still have a better ratio. These differences a larger due to the fact that H3K27ac ChIA-PET is a technique focused on H3K27ac.

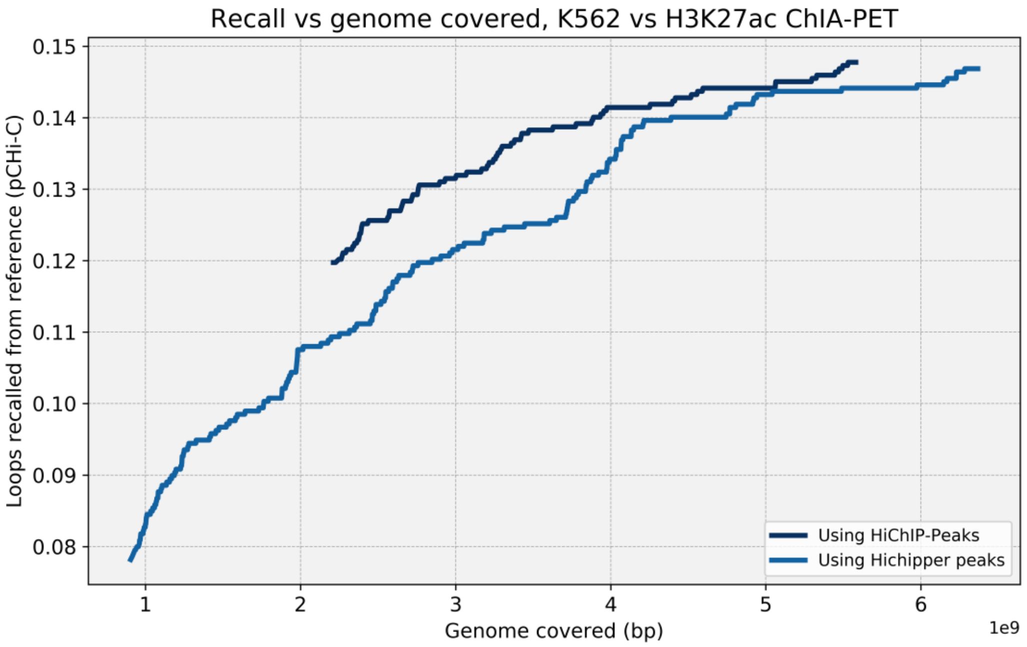

Figure S16

Overlap analysis between FitHiChIP loops called using the two different peaks sets. Fithichip shows a very high congruency of the loops regardless of the peaks used. (A) recall vs loops called compared to the other setting. (B) precision-recall plot called compared to the other setting.

A

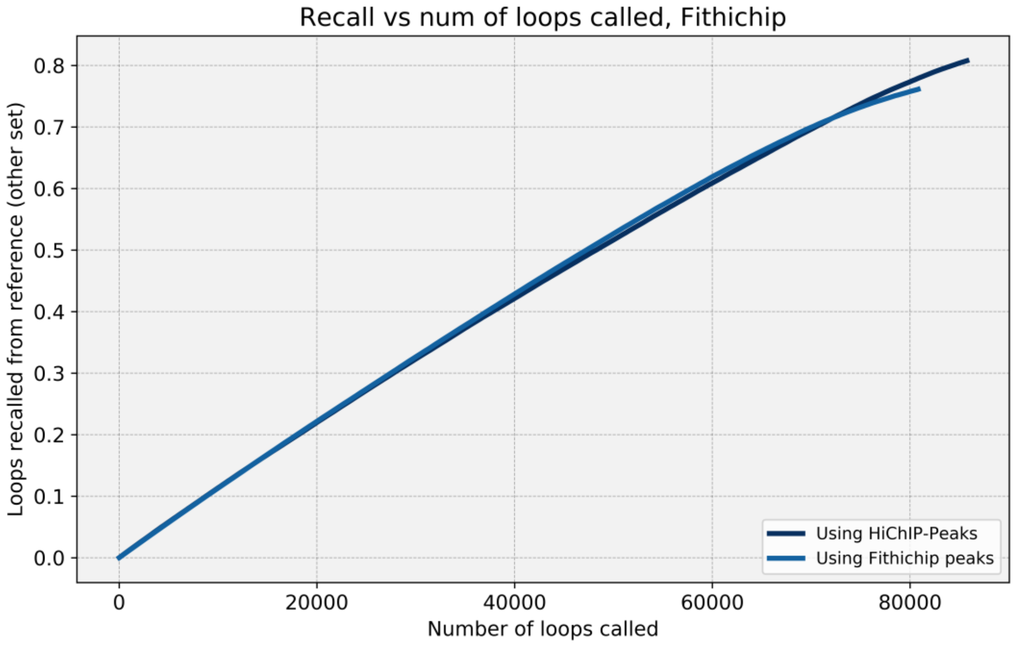

B

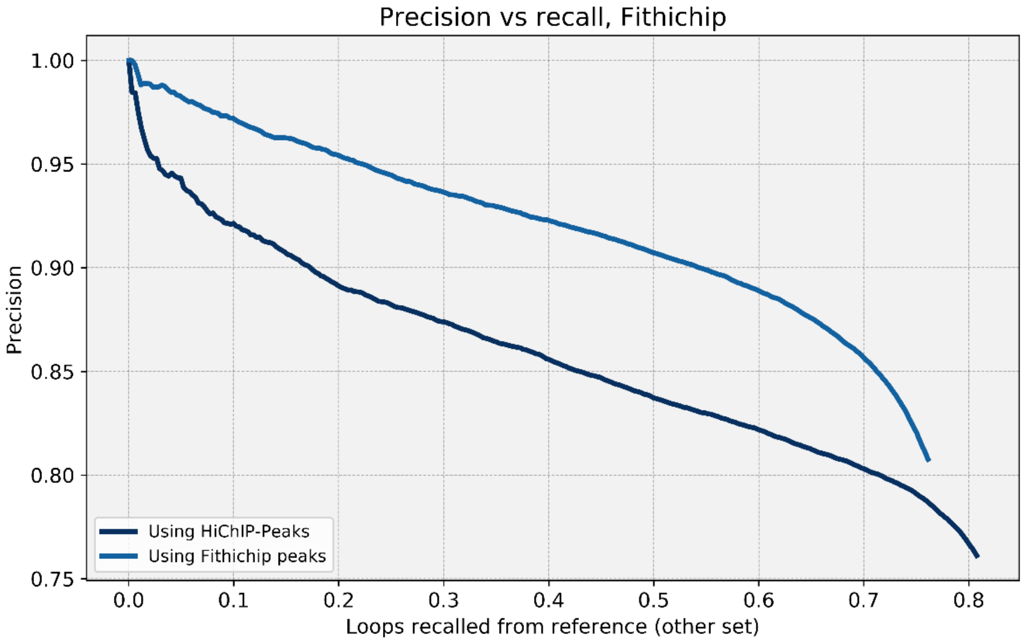

Figure S17

Overlap analysis between Hichipper loops called using the two different peaks sets. Hichipper is significantly more affected by the peaks used in the loop calling compared to FitHiChIP. (A) recall vs loops called called compared to the other setting. (B) precision-recall plot called compared to the other setting.

A

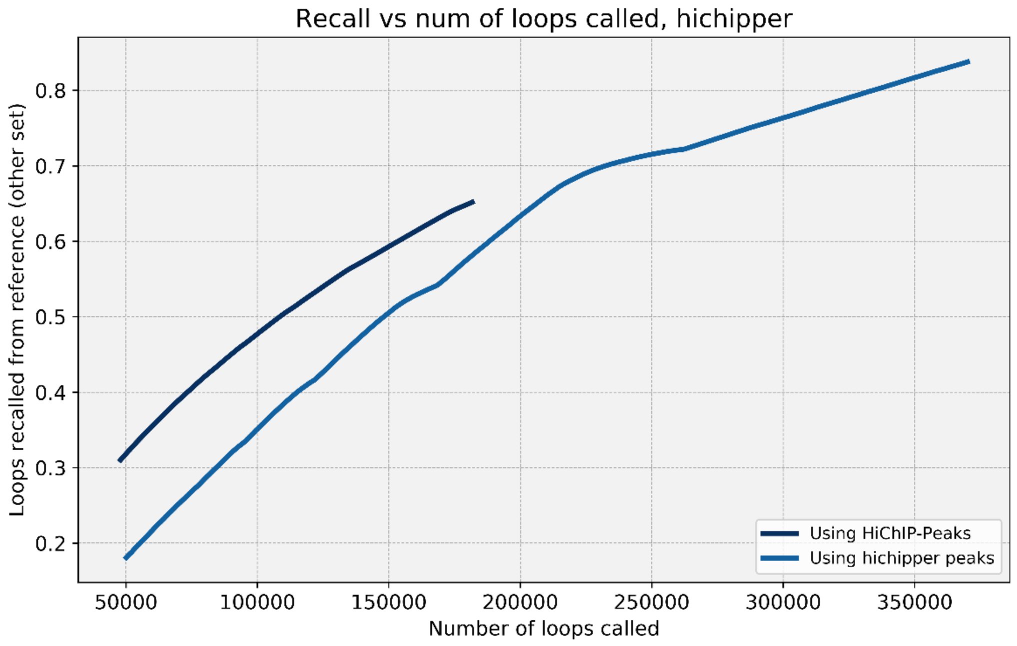

B

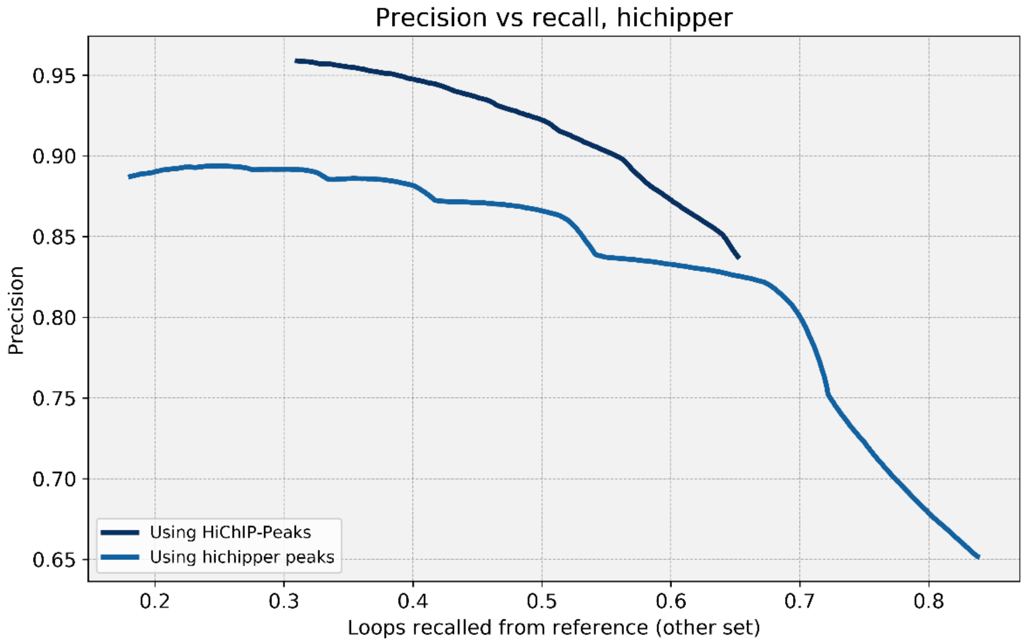

Figure S18

Overlap analysis between reference promoter capture Hi-C and FitHiChIP with default peaks and with our peaks in CD4+ Naïve T cells. Fithichip shows a very high congruency of the loops regardless of the peaks used. (A) recall vs loops called. (B) precision-recall plot.

A

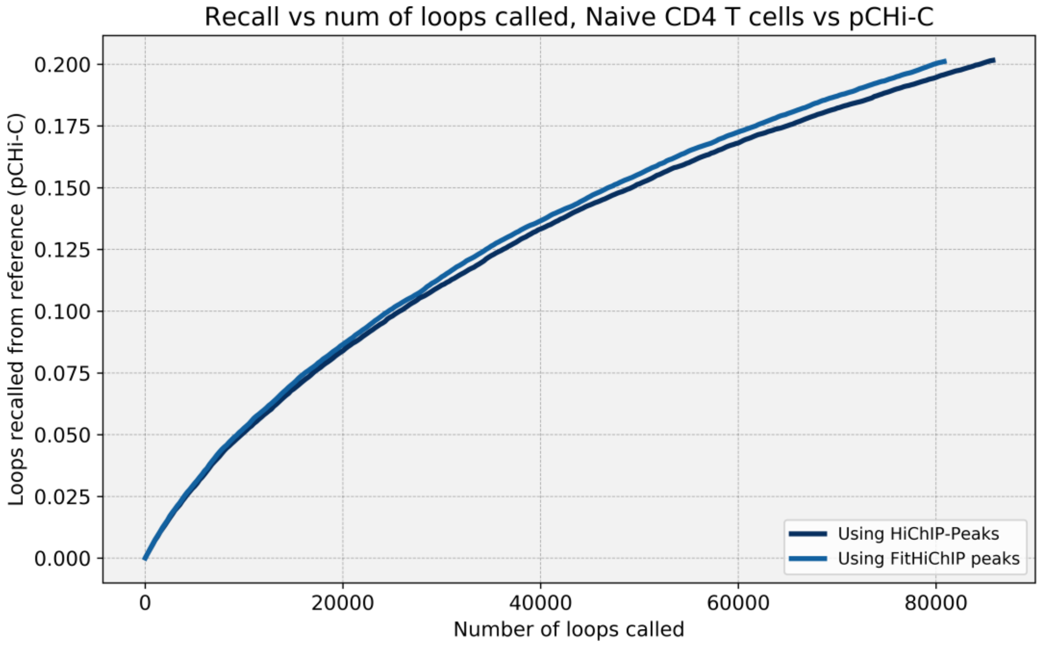

B

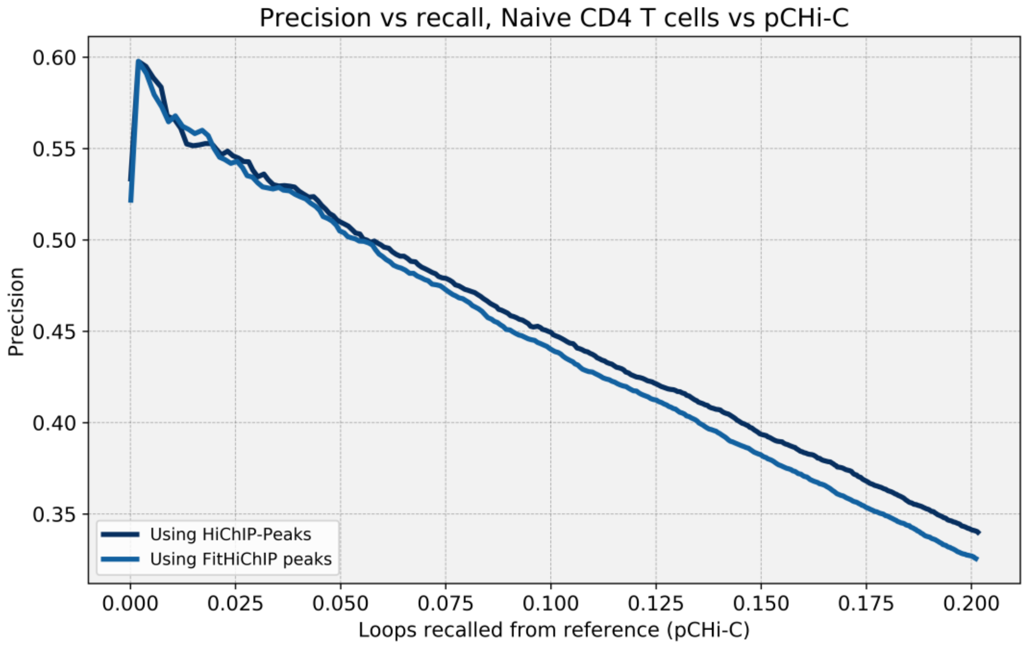

Figure S19

Overlap analysis between reference promoter capture Hi-C and FitHiChIP with default peaks and with our peaks in GM12878 cells. Fithichip shows a very high congruency of the loops regardless of the peaks used. (A) recall vs loops called. (B) precision-recall plot.

A

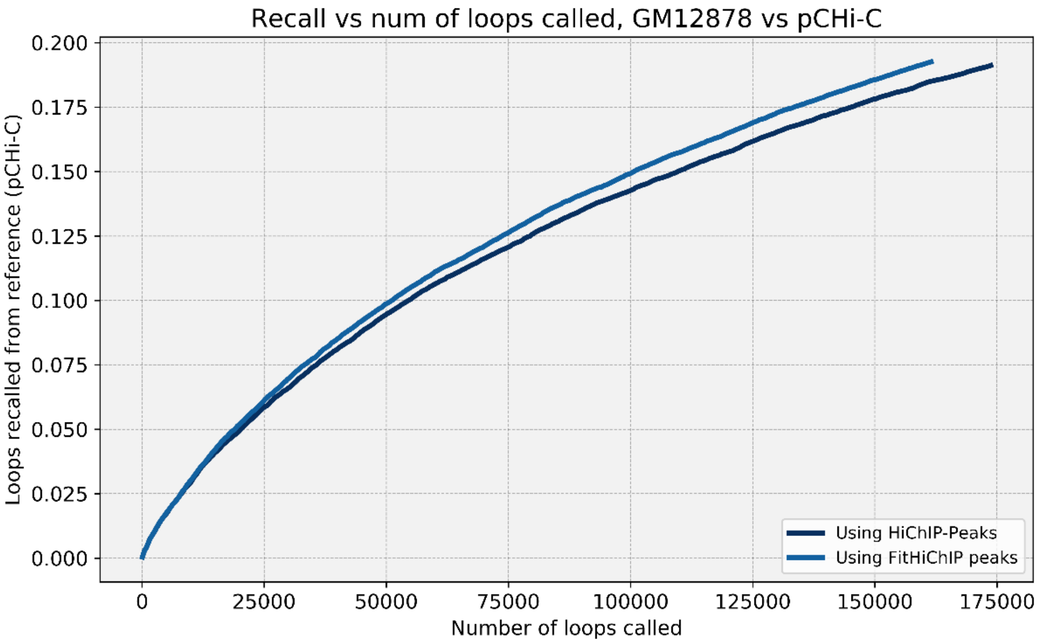

B

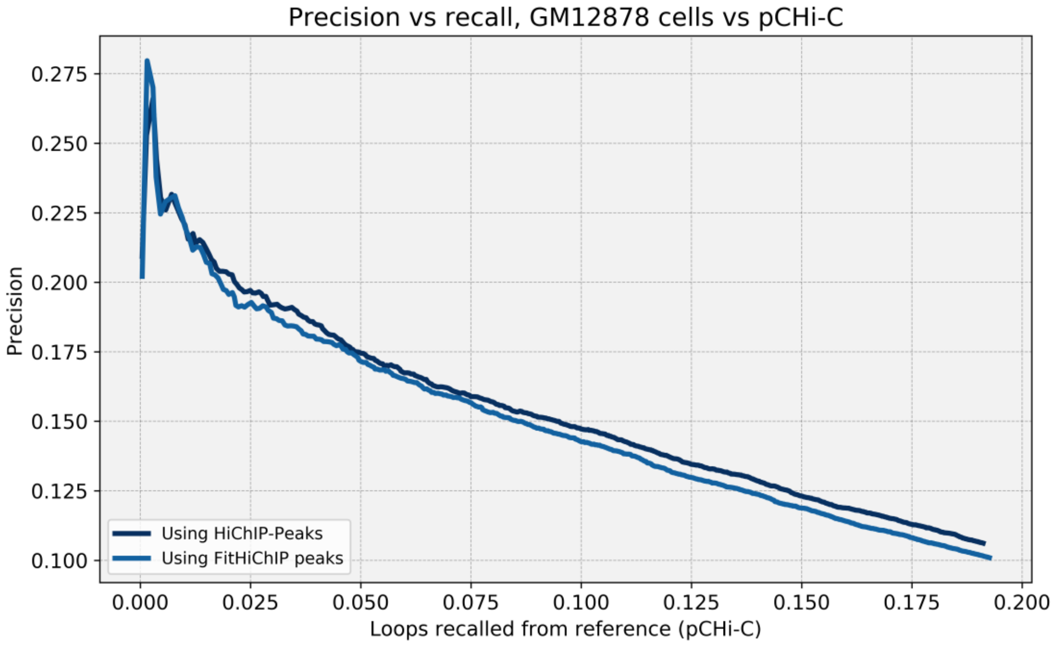

Figure S20

P-value distribution from DESeq2 for differential analysis. Naïve T cells, biological replicate B2 vs B3. The p-value distribution is as expected in the test from DESeq2, which is a U shape when lfc is set to anything different than 0. (A) lfc = 0.5 (used for the results presented in this paper). (B) lfc = 0.

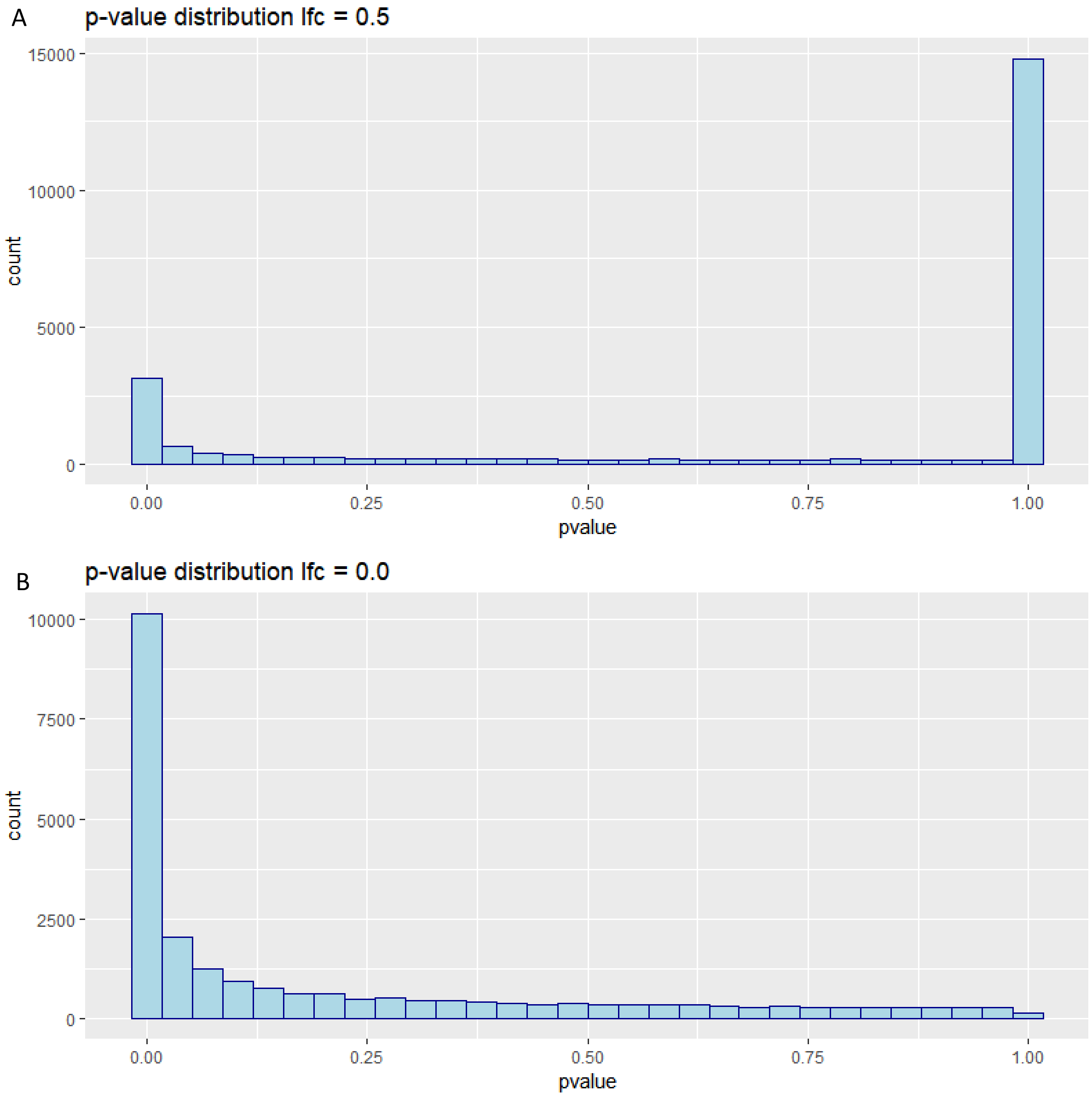

Supplement: btaa202_Supplementary_Data [file btaa202_supplementary_data.pdf]
